# The Burden of Stroke: Incidence, Hospitalizations, Mortality, and Life-Years Lost in Sweden during 2000–2022

**DOI:** 10.1159/ned/adwag027

**Published:** 2026-09-16

**Authors:** Kristian Bolin, Katharina Stibrant Sunnerhagen

**Affiliations:** a Centre for health Governance, Department of Economics, University of Gothenburg, Gothenburg, Sweden; b Department of Clinical Neuroscience, Institute of Neuroscience and Physiology, Sahlgrenska Academy, Goteborg, Sweden

**Keywords:** Stroke, Costs associated with stroke, Reperfusion therapy

## Abstract

**Background and aims:**

There are no estimates of the disease burden and economic burden of stroke in Sweden for (>2) consecutive years. The objective of this study is to derive estimates of the “annual” disease and economic burden of stroke in Sweden for the years 2000–2022.

**Methods:**

This study employs total population Swedish register data pertaining to hospital-based care, mortality and sickness-absenteeism reimbursement due to stroke for the period 2000–2022 in order to estimate the disease and economic burden of stroke. The annual economic burden of stroke was calculated for the years 2000–2022, using unit costs based on data from Statistics Sweden and the Swedish National Board of Health and Welfare, and reported in 2022 prices.

**Results:**

The incidence of stroke among males aged 50 to <60 years and 60 to <70 years decreased by about 30% and 50% between 2000 and 2022. The corresponding figures among females were estimated at about 40% and 50%. Stroke mortality among persons aged >50 years declined by >60% during the same period. The decrease in mortality corresponds to a reduction in annual expected lost working years by about 50%. The annual number of reimbursed days of sickness absenteeism is about the same at the end of the study period as at the beginning. The direct economic burden of stroke was estimated at about Eur 0.51 billion annually, while indirect costs (productivity losses) decreased from Eur 0.31 billion to Eur 0.24 billion. Thus, the total economic burden of stroke declined from Eur 0.84 billion in 2000 to Eur 0.74 billion in 2022.

**Conclusions:**

The decline in the economic burden of stroke over the study period reflects improved stroke treatment.

## Introduction

The 2021 Global Burden of Disease study concluded that the global stroke burden of disease is substantial and increased between 1990 and 2021 [1]. Globally, stroke was among the 3 most common causes of death and losses of disability-adjusted life years in 2021. More specifically, the study found that the age-standardized incidence of stroke among persons aged <55 years increased significantly over the covered period. In Europe, 1.5 million people were diagnosed with stroke, and 400,000 people died due to stroke in 2017 [2]. The total economic burden of stroke was estimated at Eur 60 billion, out of which healthcare accounted for slightly <50% [2]. A rough estimate of the economic burden of stroke in Sweden, based on these figures and the assumption that 2% of the estimated cost of stroke in Europe is due to stroke occurring in Sweden, suggests that the economic burden of stroke in Sweden in 2017 was about Eur 1.2 billion. The disease and economic burden of stroke in Sweden have been estimated in a small number of studies [3–8], in all cases for a single year or for 2 separate years (in some cases consecutive).

The aforementioned studies establish that the economic burden of stroke is considerable. However, the development of direct and indirect costs associated with stroke over time has been studied to a lesser extent. More specifically, there are no estimates of the disease burden and economic burden of stroke in Sweden (published in peer-reviewed journals) for more than 2 consecutive years using total-population patient-level data and calculated with identical methods in every year. Thus, the objective of this study is to derive methodologically consistent estimates of the “annual” disease and economic burden of stroke in Sweden for the years 2000–2022 using individual-level registry data, and to compare these trajectories with the development of measures reflecting (1) the development of stroke treatment and (2) the effectiveness of stroke treatment. These developments highlight the benefits of allocating resources to stroke-treatment advances and constitute a basis for assessing the cost-effectiveness of new treatments.

## Data and Methods

The burden of disease calculations were performed applying a prevalence approach, i.e., total disease burden and costs associated with stroke were calculated separately for each year. The calculations of productivity losses were performed somewhat differently by summarizing the total expected future value of production lost due to death in a certain year (an incidence approach).

The data employed in this study were collected from Swedish total-population individual-level registers and comprise information about hospitalizations, mortality, and reimbursed days of sickness absenteeism from work, for the years 2000–2022. More specifically, the study population was identified by the following criteria: at least one registration in the Swedish Patient Registry (Swedish National Board of Health and Welfare) with main diagnosis ICD10: I61 (intracerebral hemorrhage), I63 (cerebral infarction), or I64 (unspecified stroke). The information collected includes date of hospital admission, date of discharge, registered main diagnosis, date of death and (main) cause of death according to ICD10 (Cause of Death Register, Swedish National Board of Health and Welfare; ICD10 was introduced in Sweden in 1997), and reimbursed days of sickness absenteeism (Swedish Social Insurance Agency). The Swedish Patient Register essentially covers all hospital visits and has high diagnosis validity [9, 10]. Complementary information about the frequency of reperfusion therapy (thrombolysis and/or thrombectomy) over the aforementioned time period was collected from the Swedish Stroke Register. Demographic information was collected from Statistics Sweden.

### Swedish Institutional Context

The extent to which emergency healthcare is provided obviously determines outcomes in case of a stroke. The Swedish healthcare sector is publicly funded through taxes, but some outpatient services are privately produced. Nonemergency and elective healthcare are associated with (some) queues, but medical emergencies – such as stroke – are treated with delays almost entirely explained by patient delay or geographical factors [11]. More specifically, thrombolysis was introduced in Sweden in 2003 and gained substantial spread after 2010. Similarly, thrombectomy was first performed in 1994, but gained substantial spread after 2015.

### Demographic and Economic Changes

Demographic changes such as an increased number of elderly individuals will affect the incidence of stroke, and economic factors such as the value of time supplied to the labor market determine the value of expected working time lost in case of death or sickness absenteeism. The average age of men and women, respectively, in the Swedish population increased somewhat from 39 and 42 in 2000 to 41 and 43 in 2022. During the same period, the expected length of life increased from 72 and 77 in 2000 to 83 and 86 in 2022. The value of time measured by average hourly wage rates rose from Eur 20 and 29 in 2000 to Eur 30 and 38 in 2022 (in 2022 prices).

## Data Analysis

For the identified population, we calculated both physical and economic estimates of the societal burden of stroke. In addition, frequencies of reperfusion therapy and per-patient outcomes that reflect stroke-treatment effectiveness and its development over time, respectively, were calculated. More specifically, we calculated per 100,000 person-years, separately for the years 2000 through 2022, and by sex and age (<40, 40 to <50, 50 to <60, 60 to <70 years), the (1) number of stroke patients and nonhospital deaths (hospitalized patients plus patients registered in the Cause of Death Register with cause of death being stroke but have no registered hospital visits or hospitalizations), (2) number of ischemic and hemorrhagic stroke cases, respectively (hospitalized patients and patients dying without hospital treatment, (3) mortality rate due to stroke, and (4) mortality rates due to ischemic and hemorrhagic stroke, respectively. Moreover, the following per-patient/average outcomes were calculated: (5) share of hospitalized patients (ischemic stroke) who receive reperfusion therapy, by sex, and (6) per case/patient direct and indirect costs. (The online supplementary [for all online suppl. material, see https://doi.org/10.1159/ned/adwag027] reports (1) annual number of hospitalizations due to stroke, by sex, (2) annual number of expected working years and life-years lost, respectively, by sex, (3) annual number of reimbursed days of sickness absenteeism, (4) annual number of hospital days [contingent on hospitalization], (5) annual absolute risk of dying from stroke if hospitalized, by sex [<80 years of age], and (6) annual total direct and indirect costs, respectively, by sex.)

There are no published hospitalization unit costs for the entire studied period. Therefore, we have used statistics regarding total expenses for hospitalizations for the years 2001 (2000 figures are not readily available) through 2022 (National Accounts; Statistics Sweden) together with the total number of hospital days for the same period (Sweden National Board of Health and Welfare) to calculate annual average (all causes) cost per hospital day. Then, assuming that the development of cost per hospital day due to stroke is the same as the aforementioned development of average cost per hospital day, we calculated annual nominal costs per hospital day due to stroke using reported costs per hospital day due to stroke (the Swedish Cost-per-patient database; Swedish Municipalities and Regions, SKR) in 2022. Finally, these costs were inflated to 2022 prices using the consumer price index for healthcare goods and services (Statistics Sweden) for the studied period.

Expected life-years lost were calculated using Statistics Sweden’s length of life statistics. The corresponding productivity losses were calculated adopting the human-capital method [12], which amounts to calculating the expected gross forgone earnings over the expected lost lifetime, using observed average earned income adjusted for labor taxes (the total value of time supplied to market production includes labor taxes) reported by Statistics Sweden. More specifically, productivity losses were calculated separately for men and women and for individuals in the age span of 16–70 years. For an individual dying from stroke at *x* years of age, the expected lost value of production was calculated using information on expected remaining lifetime at *x* years and data on incomes per 1-year age groups for ages beyond *x*. Productivity losses due to sickness absenteeism from work were estimated analogously by first calculating productivity losses per day, assuming that annual incomes are earned during 212 annual workdays. Calculations were performed for the years 2005–2022 (the diagnosis reporting in the register administered by the Swedish Social Insurance Agency is unreliable and/or missing before 2005). In both cases, average incomes for the different years were inflated to 2022 prices using the consumer price index.

## Results

### Total Population

For the period 2000 through 2022, 476,459 patients were hospitalized due to stroke on 720,318 different occasions. Over the same period, 75,966 hospital-treated patients and 30,381 individuals who had no hospital-based healthcare contact due to stroke died from stroke. Table 1 describes this population jointly for the entire period.

**Table 1.** Aggregate statistics for the period 2000–2022

​	No. of unique patients (% men)	No. of hospitalizations (% men)	Average no. of hospital days, men (women) (among hospitalized)	No. of deaths, men (women)^a^	No. of lost expected life-years^b^	No. of days of sickness absenteeism^c^
<40	8,438 (54%)	10,699 (56%)	12.1 (12.4)	117 (70)	5,732 (3,831)	1,497,585
40–50	14,592 (61%)	18,916 (63%)	12.7 (11.8)	402 (188)	13,972 (7,399)	3,528 250
50–60	42,355 (65%)	55,406 (67%)	12.2 (11.7)	1,475 (804)	38,177 (24,112)	9,009,929
60–70	90,827 (64%)	119,456 (65%)	11.1 (10.7)	4,206 (2,401)	73,430 (50,423)	5,250,993
70–80	159,590 (56%)	212,561 (56%)	10.4 (10.8)	11,650 (9,116)	120,434 (116,959)	54,747
80–90	179,067 (43%)	239,875 (43%)	10.8 (11.4)	20,043 (28,179)	105,524 (188,612)	2,847
>90	51,254 (30%)	63,405 (30%)	9.9 (10.3)	7,636 (20,060)	18,608 (62,262)	–

Number of unique patients hospitalized due to stroke, number of hospitalizations, average number of hospital days, and lost expected life-years.

^a^ Sum of deaths occurring in hospital and deaths occurring without hospitalization.

^b^ Expected lost life-years are calculated for deaths occurring at ≤100 years of age.

^c^ Number of days reimbursed by the social security system plus adjustment for days not reimbursed. Every case of uninterrupted absenteeism is reimbursed by the social security system from and including day 15. Thus, for every such case, the total number of sickness absenteeism days is 14 + the number of reimbursed days.

### Annual Disease and Economic Burden of Stroke

The statistics reported in Table 1 clearly imply that the disease and, consequently, the economic burden of disease associated with stroke is considerable. The development of the disease burden of stroke over the period 2000 through 2022 is illustrated in Figures 1–8. The annual number of stroke patients and nonhospital deaths (per 100,000 person-years) declined among those aged 50 years or older over the study period. The most pronounced decline was among persons aged 60–70 years. Between 2000 and 2022, the decline was about 50% for both men and women, from about 700 to 350 among men and from about 375 to 200 among women (Fig. 1, 2). The corresponding figures for ischemic and hemorrhagic stroke largely mirror the figures for stroke total (Fig. 3–6). The development among those aged ≥70 years shows about the same relative change (not shown in the figures). The average length of hospitalization due to stroke declined markedly from about 14 days for both men and women in 2000 to about 8 days for both sexes in 2022.

**Fig. 1.**
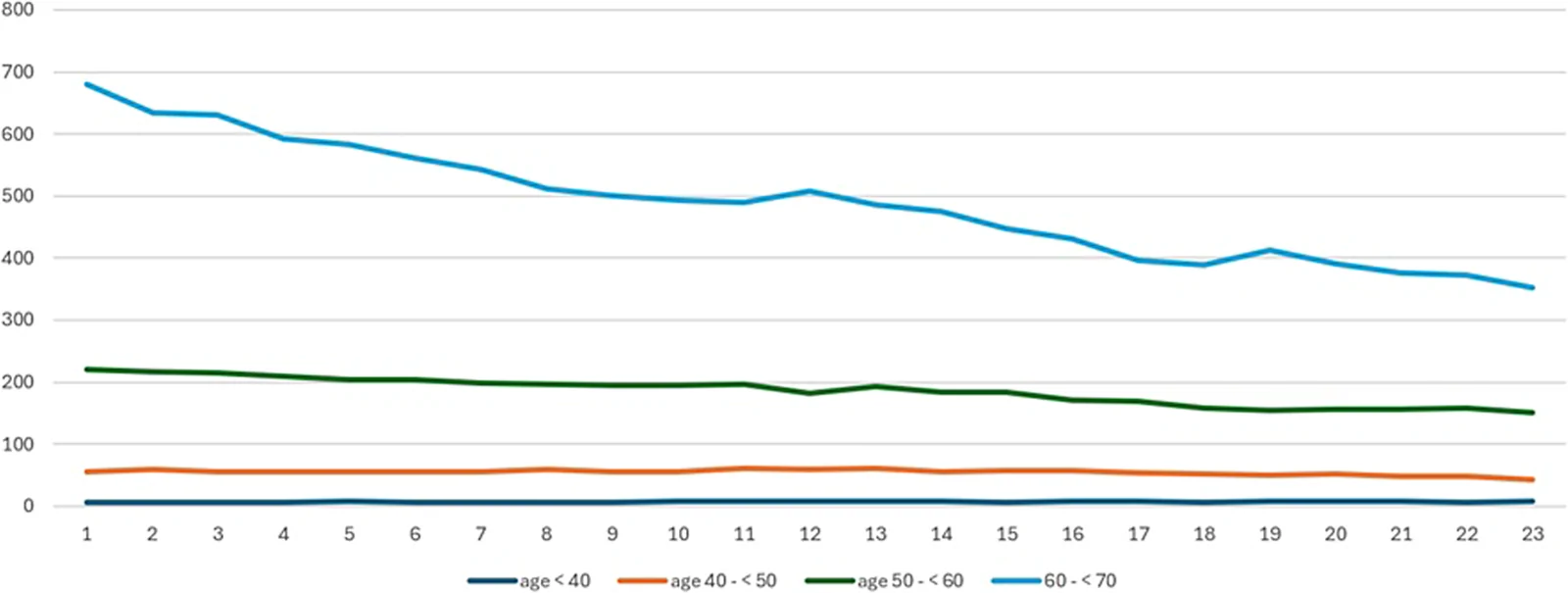
Annual number of stroke patients/cases (hospitalized plus dying without hospitalization) per 100,000 person-years, by age group, men.

**Fig. 2.**
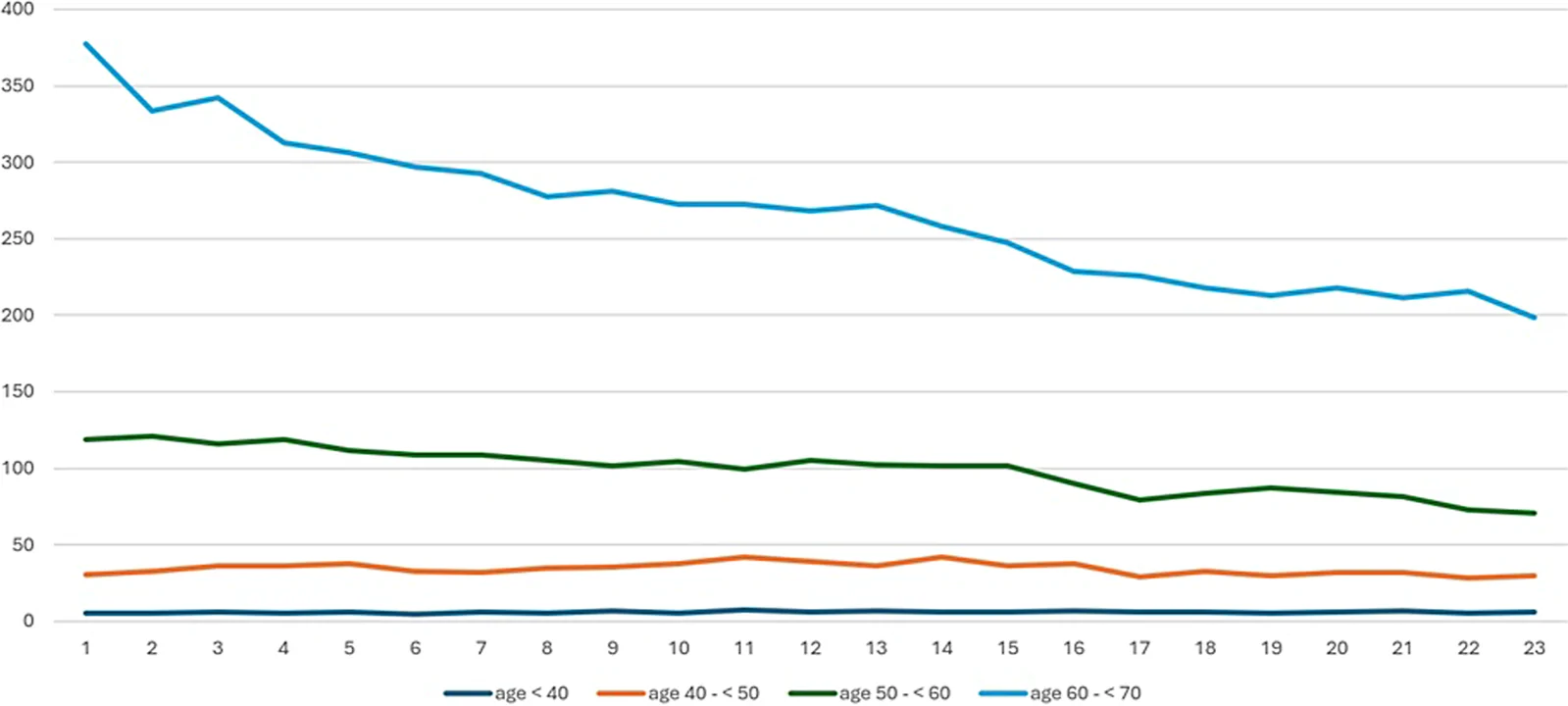
Annual number of stroke patients/cases (hospitalized plus dying without hospitalization) per 100,000 person-years, by age group, women.

**Fig. 3.**
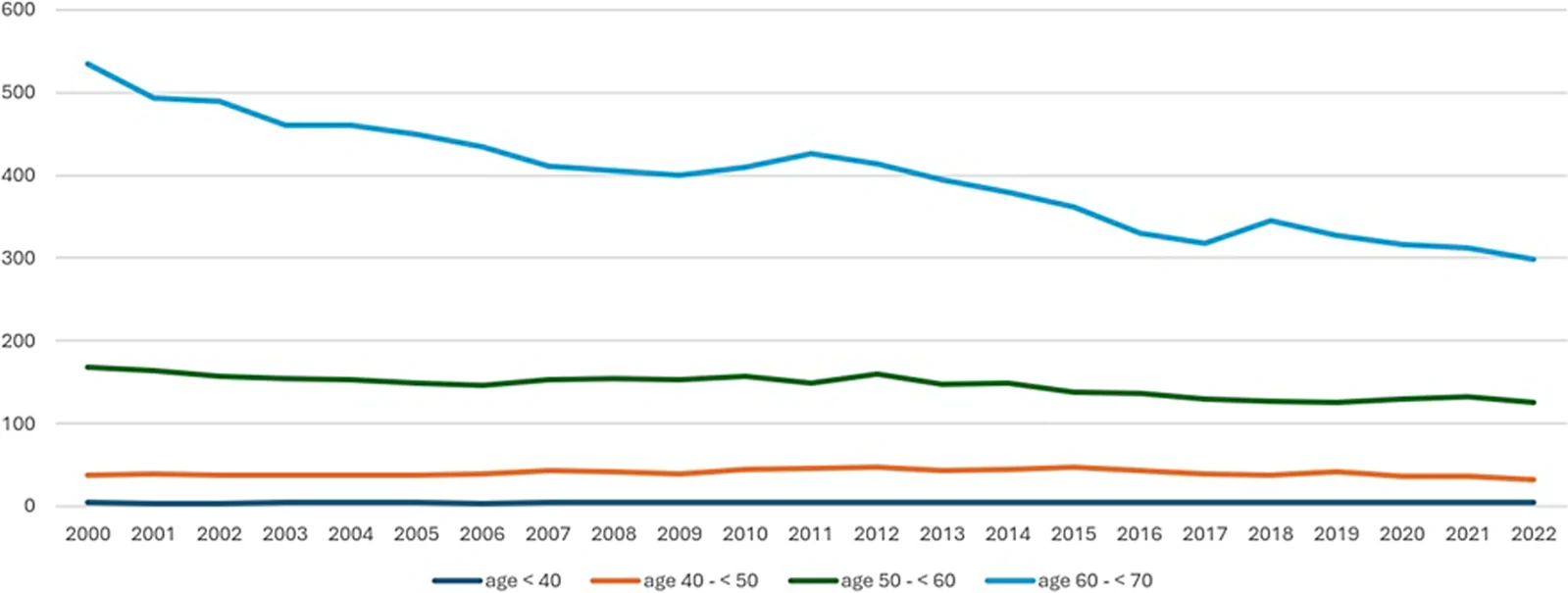
Annual number of ischemic stroke patients (hospitalized plus dying without hospitalization) per 100,000 person-years, by age group, men.

**Fig. 4.**
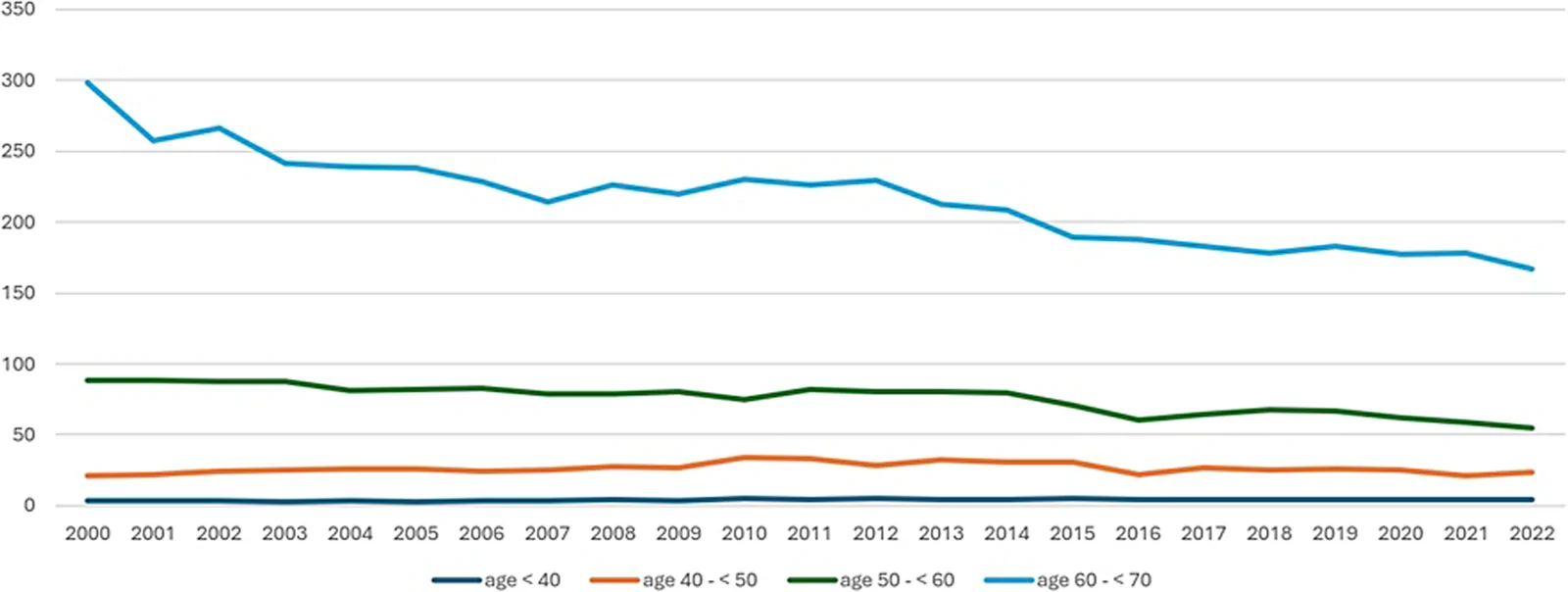
Annual number of ischemic stroke patients (hospitalized plus dying without hospitalization) per 100,000 person-years, by age group, women.

**Fig. 5.**
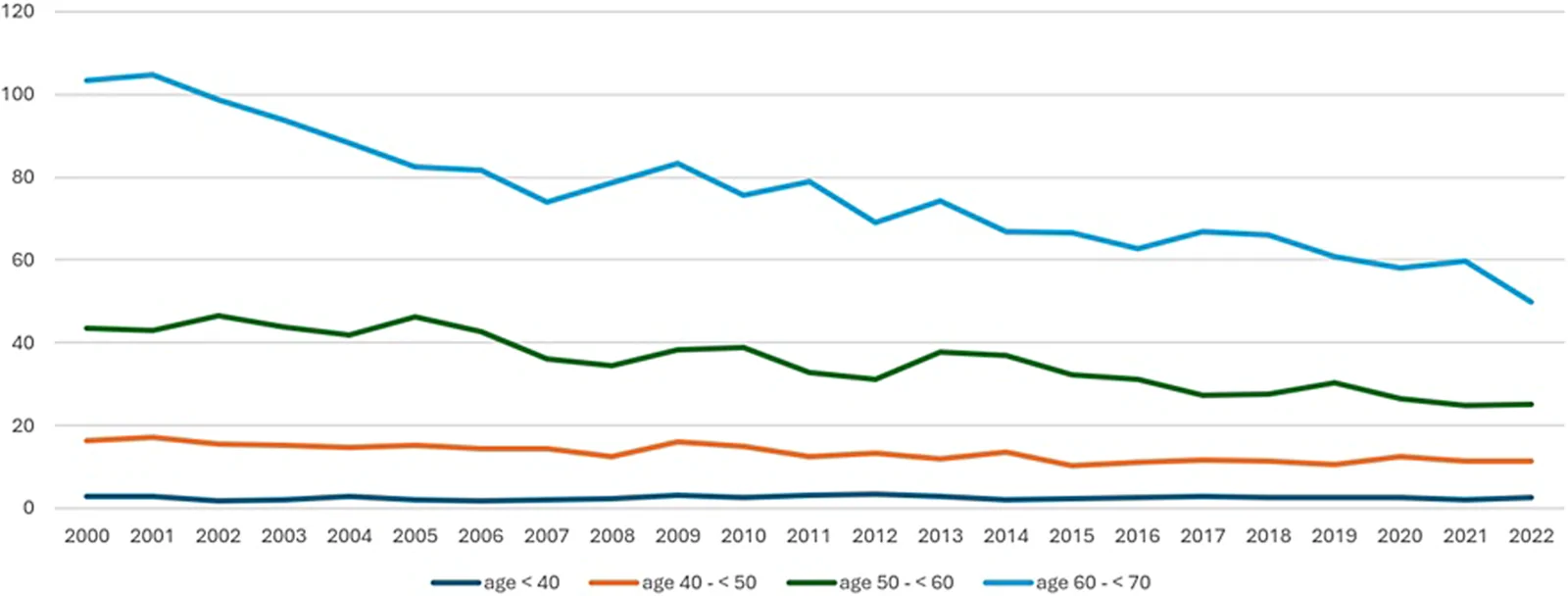
Annual number of hemorrhagic stroke patients (hospitalized plus dying without hospitalization) per 100,000 person-years, by age group, men.

**Fig. 6.**
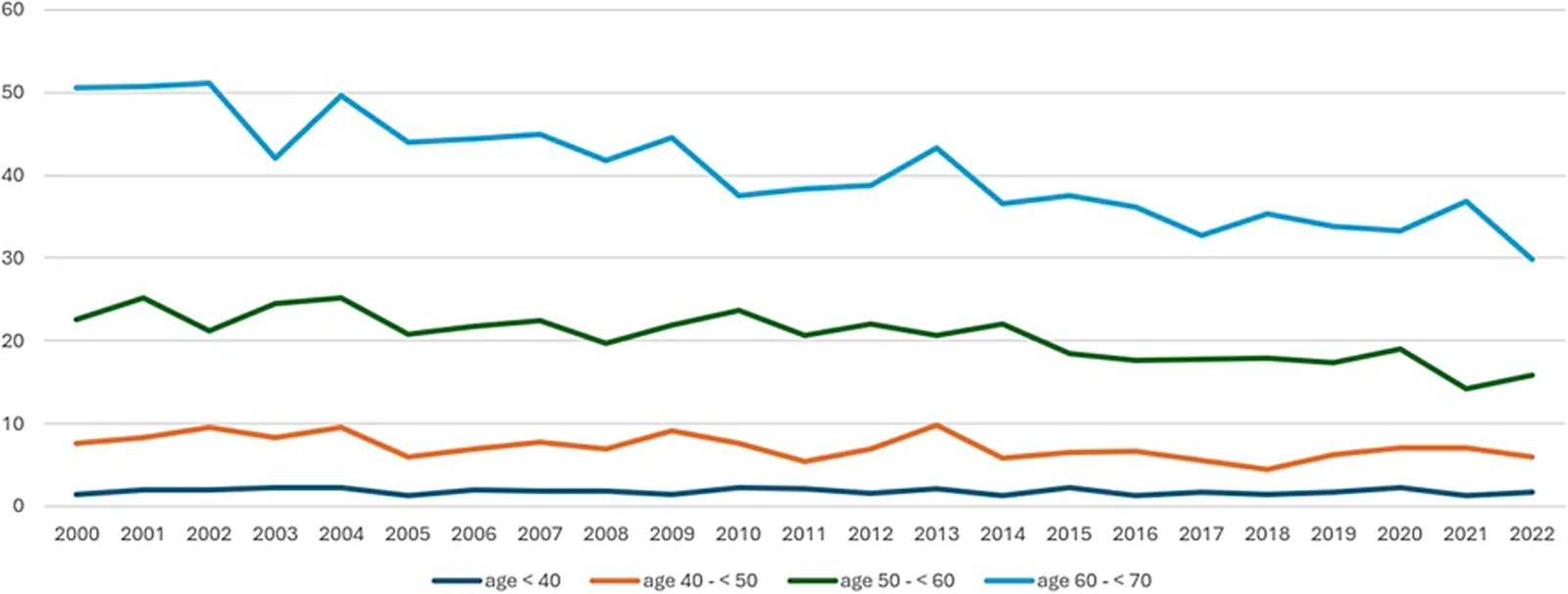
Annual number of hemorrhagic stroke patients (hospitalized plus dying without hospitalization) per 100,000 person-years, by age group, women.

**Fig. 7.**
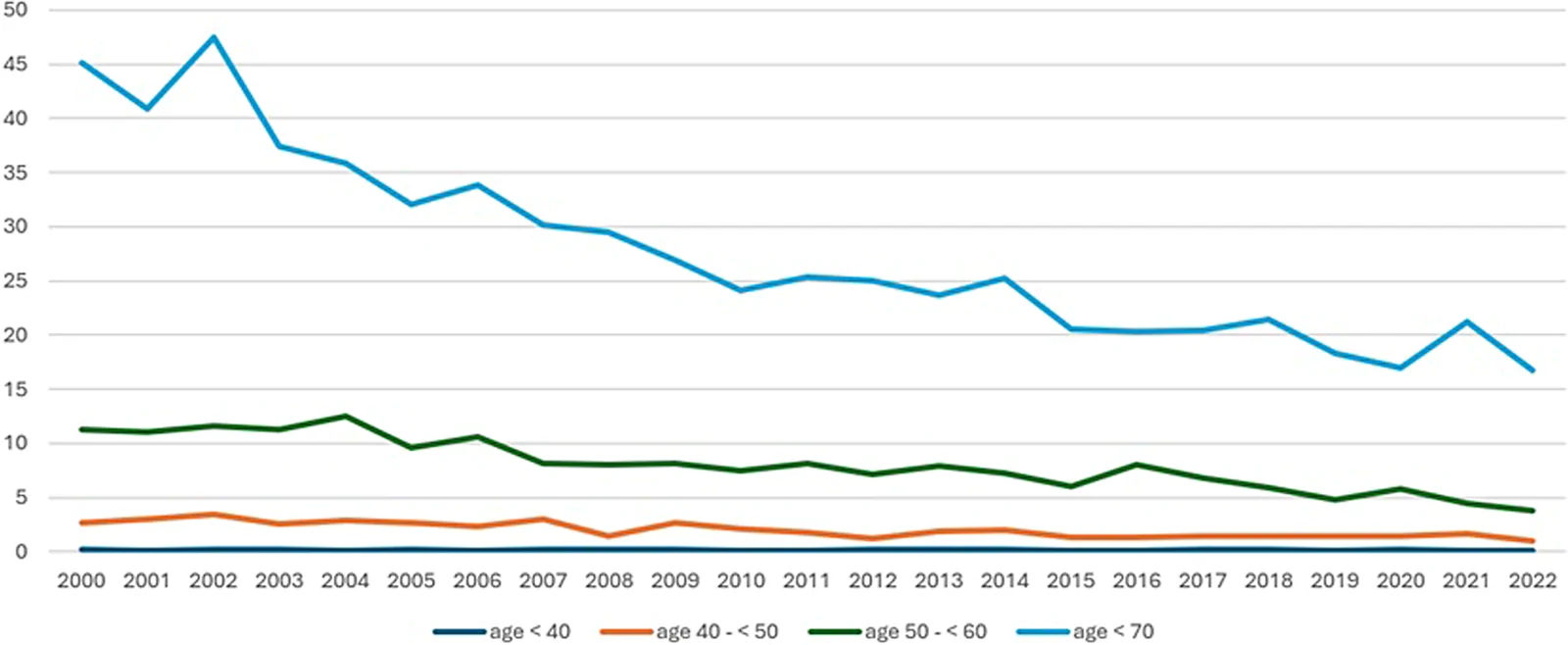
Annual mortality rate due to stroke, by age group (<70), per 100,000 person-years.

**Fig. 8.**
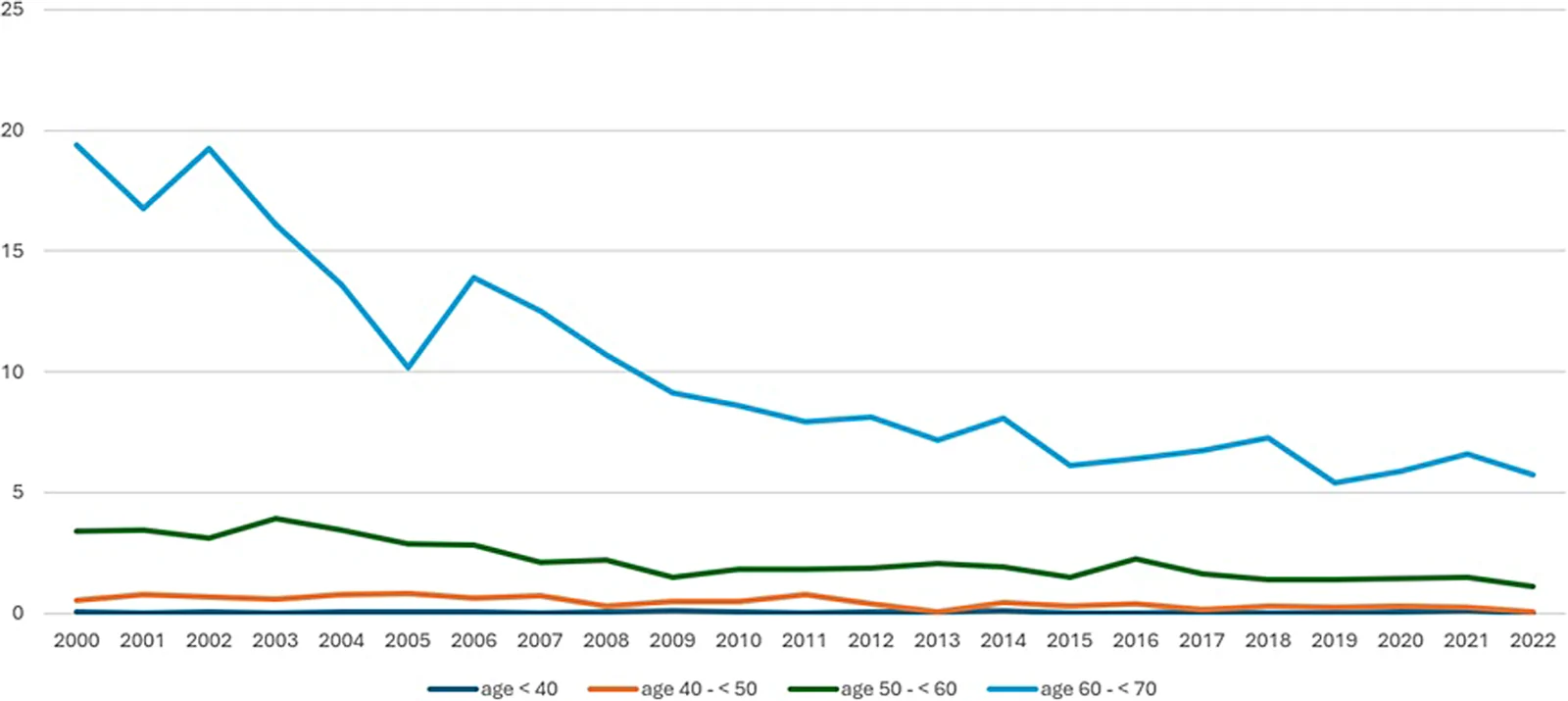
Annual mortality rate due to ischemic stroke, by age group (<70), per 100,000 person-years.

Mortality rates for stroke in the age group 60–70 years of age were estimated at about 45 per 100,000 person-years in 2000 and about 16 in 2022 (Fig. 7). The corresponding figures for the subgroups ischemic stroke and hemorrhagic stroke mortality (Fig. 8, 9), respectively, were estimated at about 20 and 18 cases in 2000 and about 6 and 7 cases in 2022. The absolute risk of dying (among hospitalized patients aged <80 years) declined from approximately 8% in 2000 to about 6% for men and slightly <7% for women in 2022 (online suppl.). These figures translate into the following changes in lost expected working years and life-years (online suppl.). Stroke mortality in 2000 resulted in about 7,500 and 5,000 expected lost working years (men, women) and about 28,000 and 22,000 lost expected life-years. The corresponding figures due to stroke mortality in 2022 had declined to about 3,200 and 2,100 lost expected working years and 13,000 and 12,000 lost expected life-years in 2022 (online suppl.).

**Fig. 9.**
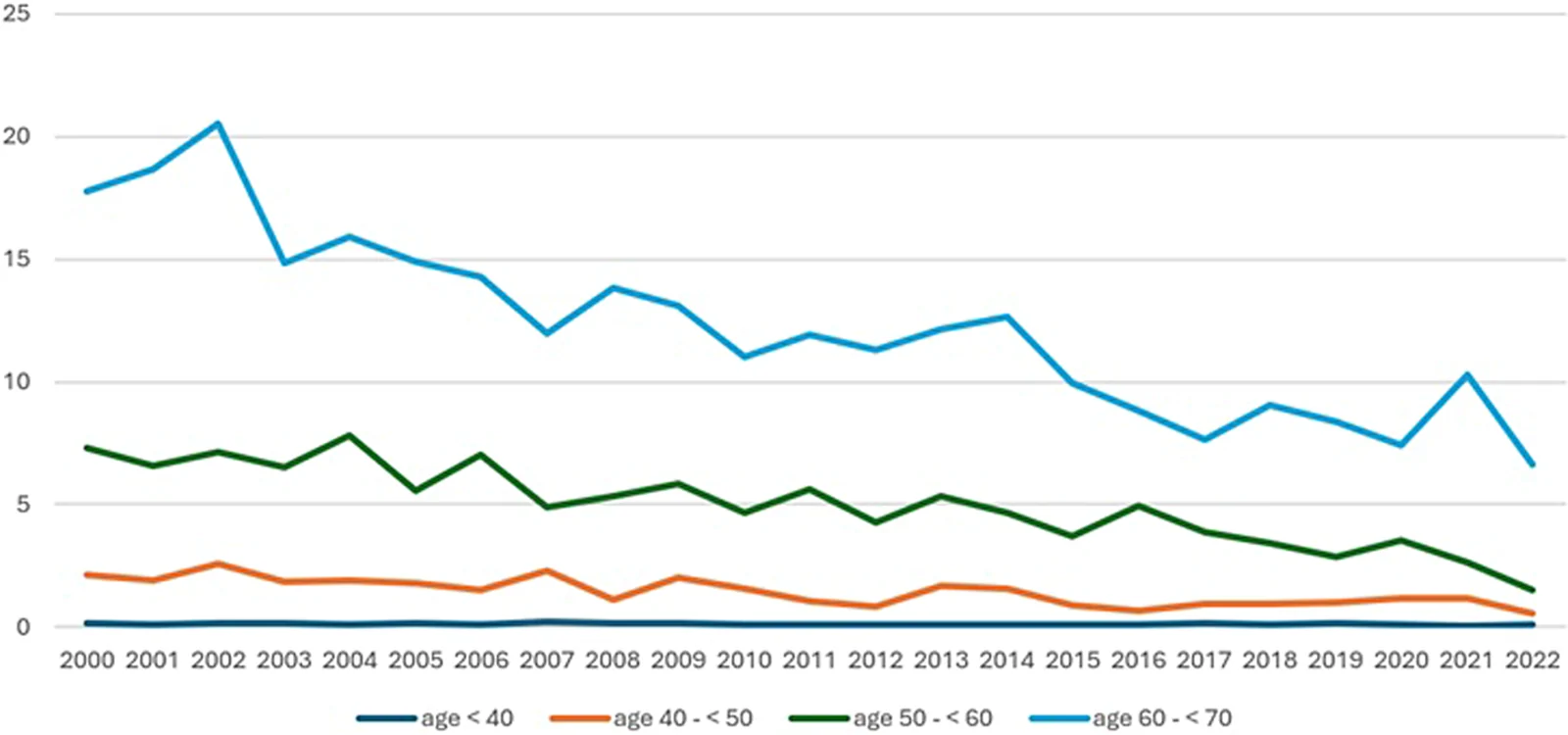
Annual mortality rate due to hemorrhagic stroke, by age group (<70), per 100,000 person-years.

Annual relative frequencies of ischemic stroke patients receiving reperfusion therapy are illustrated in Figure 10. In 2003, <2% of (hospitalized) ischemic stroke patients received reperfusion therapy. The corresponding figure in 2022 was about 12% for both men and women.

**Fig. 10.**
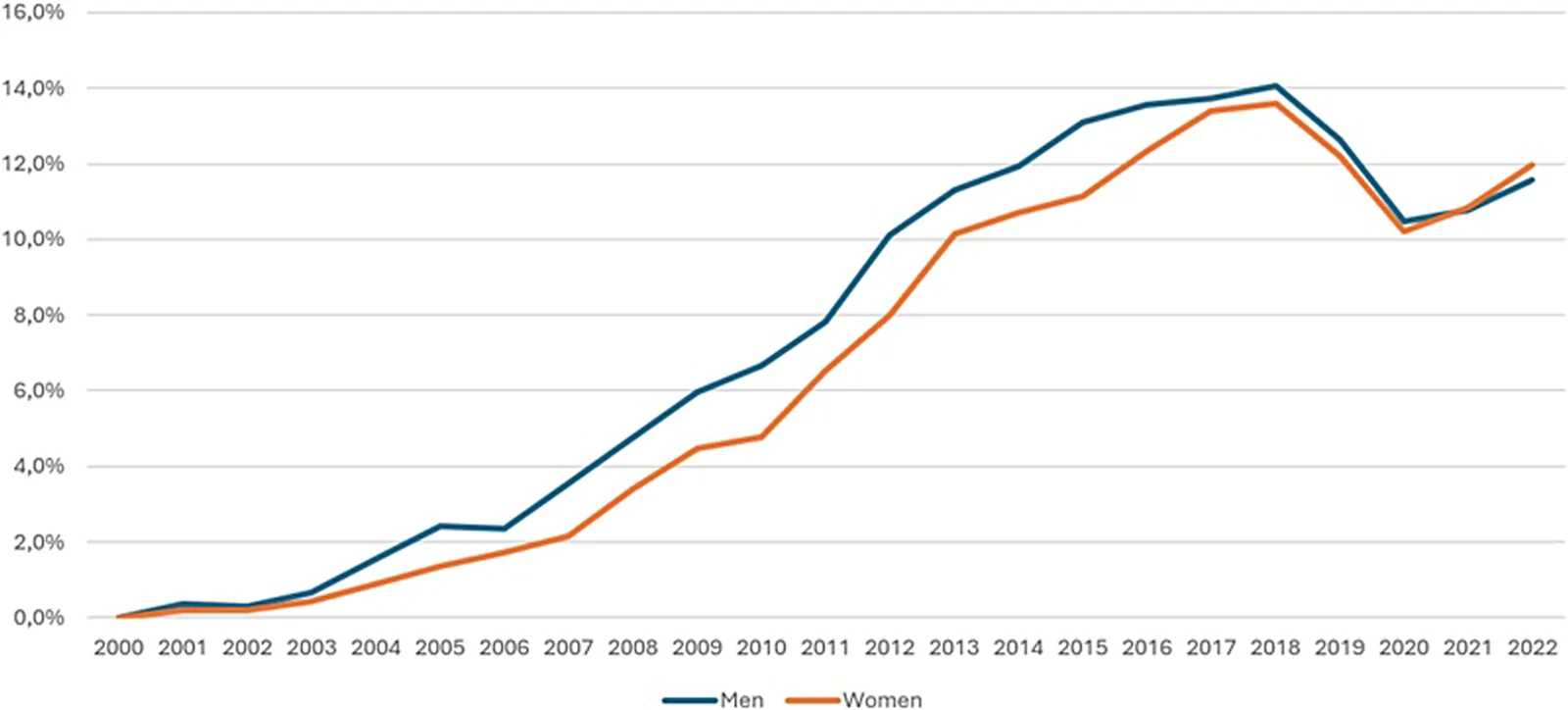
Average annual number of patients receiving reperfusion therapy (as shares of the number of ischemic stroke patients), by sex.

The per-patient economic burden associated with stroke treatment (hospitalizations) and mortality and sickness absenteeism is illustrated in Figures 11 and 12 (the numerical values producing Fig. 1–12, and total direct and indirect costs are reported in the online suppl.).

**Fig. 11.**
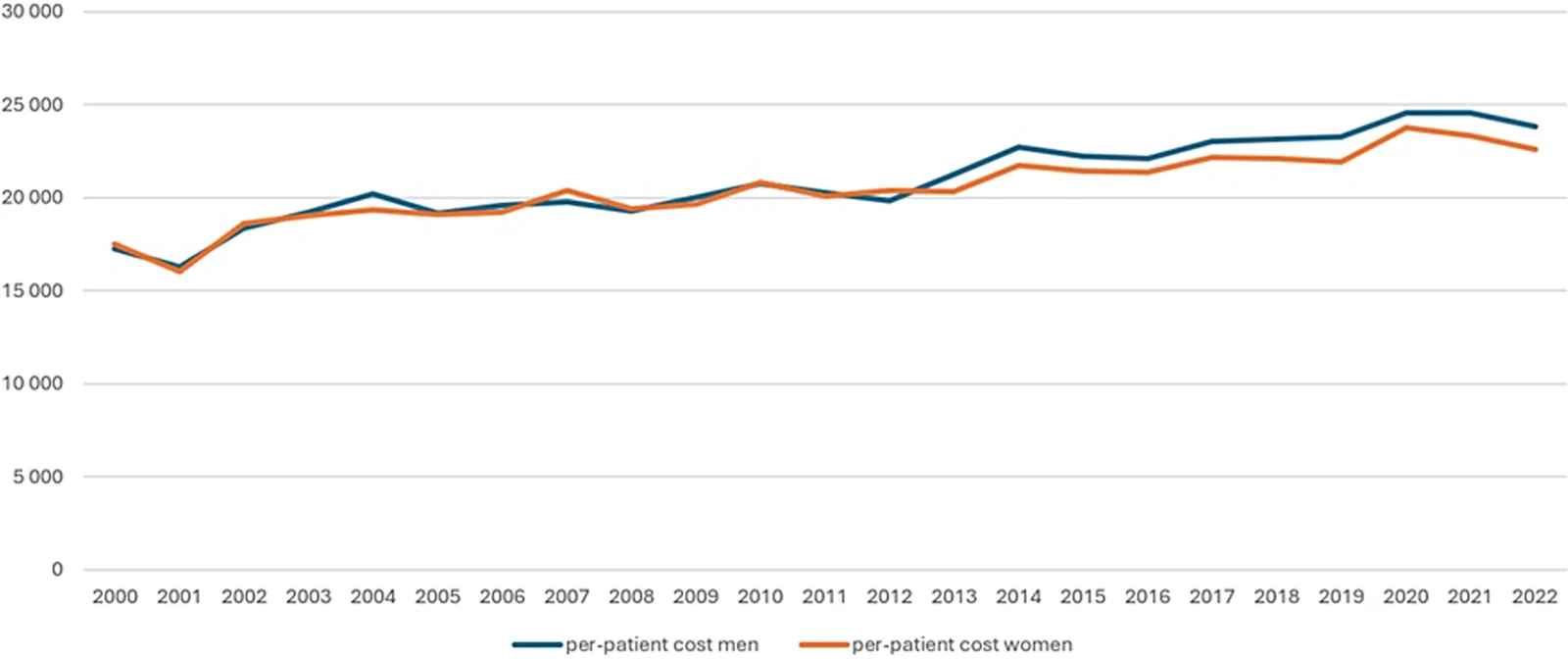
Annual per patient direct costs (hospitalizations) due to stroke, by sex, 2022 prices.

**Fig. 12.**
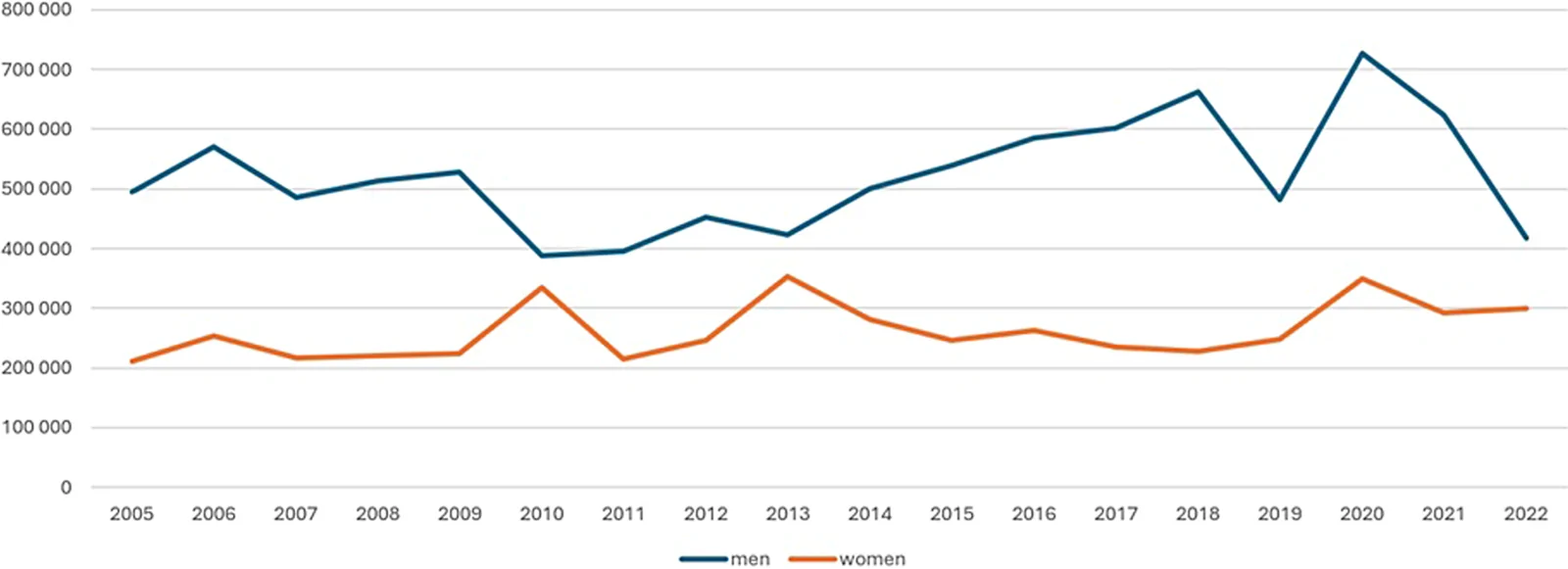
Annual per patient indirect costs (Eurs, productivity losses due to mortality and sickness absenteeism) due to stroke, 2022 prices.

### Cost Associated with Stroke

All monetary values are reported in 2022 prices. Annual per-patient direct costs rose somewhat from Eur 17,000 for both men and women in 2000 to about Eur 23,000 for men and Eur 22,000 for women in 2022. Total direct costs were calculated at about Eur 506 million annually (online suppl.). Indirect costs due to stroke showed a somewhat different time pattern. The costs associated with stroke mortality and sickness absenteeism among men were significantly higher than the corresponding costs among women. Per-case indirect costs associated with stroke mortality in 2005 were estimated at about Eur 500,000 among men and Eur 200,000 among women, while the corresponding costs in 2022 were estimated at Eur 400,000 and Eur 300,000, respectively. Mortality and sickness absenteeism in 2005 induced total indirect costs at about Eur 310 million (online suppl.). By 2022, the total indirect costs had declined to about Eur 240 million. Over the study period, the total economic burden of stroke declined somewhat from about Eur 844 million in 2005 to Eur 741 million in 2022 (as mentioned above in the Data analysis subsection, data on diagnosis underlying reimbursement for sickness absenteeism are unreliable before 2005).

## Discussion and Conclusion

In this study, the disease and economic burden associated with stroke was calculated separately for the years 2000 through 2022 employing total-population individual-level registry data. In summary, we found that during the study period 2000–2022 the number of stroke patients hospitalized or dying without hospitalization per 100,000 person-years declined markedly among persons aged >50 years, while small or insignificant changes were observed for the younger age groups. The same pattern was observed for the subgroups ischemic stroke and hemorrhagic stroke, with a marginally less pronounced decline in the latter group. Stroke mortality decreased markedly over the study period. Total stroke mortality decreased by about 64%, while mortality due to ischemic stroke decreased by 70%.

While the per-patient direct cost of stroke rose over the study period, the direct economic burden of stroke (in 2022 prices) was roughly constant over the studied period (online suppl.). No clear trends could be observed in per-patient or total indirect costs, although total indirect costs were lower in 2022 than in 2000. However, the decline in the disease burden of stroke – the decline in the number of deaths due to stroke, the shift in the age distribution of stroke mortality towards older individuals (see per-patient expected losses of working years and life-years, online suppl.), and the decline in the average duration of stroke hospitalization (online suppl.) – suggests that the effectiveness of stroke treatment, and perhaps stroke prevention, has improved over the studied period. Thus, even though the economic costs attributable to stroke are relatively stable over time in real (2022) prices, the adverse effects have declined over time, suggesting that the cost-effectiveness of stroke care has improved over the studied period.

It seems plausible that the decline in the risk of dying from stroke (if hospitalized), the shift in the age distribution of patients dying towards older patients, and the decreased average length of hospital stay is due to more effective diagnosis and treatment. Over the studied period, a greater range of anticoagulant drugs became available, allowing for more individually adapted therapies [13–15].

## Limitations

The calculations do not distinguish between (1) first and recurrent cases and (2) different levels of stroke severity at admission. Further, costs pertaining to stroke rehabilitation are only included as far as being part of hospital-based healthcare. Finally, we do not specifically analyze the impact of the COVID pandemic on stroke-treatment outcomes.

## Statement of Ethics

This study has been approved by the Swedish Ethical Review Authority (reference number: 8866/2023; date of approval: February 01, 2024). Utilization of data from national registers, including quality registers, is exempt from the requirement of informed consent from participants (the Personal Data Act, Swedish law number SFS 1998:204).

## Conflict of Interest Statement

Kristian Bolin and Katharina Stibrant Sunnerhagen have no conflicts of interest to declare.

## Funding Sources

This study was funded by Lundbergstiftelsen and by Swedish governmental funding of clinical research (ALF) to Katharina Stibrant Sunnerhagen.

## Author Contributions

K.B. and K.S.S. jointly planned and performed this study. K.B. and K.S.S. wrote and reviewed the manuscript.

## Data Availability

Data were based on Swedish National Registers, and individual-level data cannot be shared due to national regulations. Original data are available upon application to the relevant authorities.
